# The accuracy of acetabular cup placement in primary total hip arthroplasty using an image-free navigation system

**DOI:** 10.1186/s12891-021-04902-5

**Published:** 2021-12-04

**Authors:** Yohei Naito, Masahiro Hasegawa, Shine Tone, Hiroki Wakabayashi, Akihiro Sudo

**Affiliations:** Department of Orthopaedic Surgery, Mie University Graduate School of Medicine, 2-174, Edobashi, Tsu, Mie 514-8507 Japan

**Keywords:** Image-free navigation, Total hip arthroplasty, Acetabular cup

## Abstract

**Background:**

Intraoperative navigation systems have been shown to improve the accuracy of acetabular component insertion in total hip arthroplasty (THA). The purpose of this study was to investigate the accuracy of cup orientation in primary THA using an image-free navigation system.

**Methods:**

A total of 107 consecutive cementless THAs using an image-free navigation system were performed from February 2017 to March 2020 (the navigation group). As a control group, 77 retrospective consecutive cases who underwent THAs with manual implant-techniques between February 2012 and April 2017 were included. Postoperative cup radiographic inclination and radiographic anteversion relative to the functional pelvic plane were assessed using a 3D-template system after computed tomography (CT) examination.

**Results:**

The mean absolute errors of the postoperative measured angles from the target angles in inclination were 3.4° ± 3.0° in the navigation group and 8.4° ± 6.6° in the control group (*p* < 0.001). The mean absolute errors in anteversion were 5.1° ± 3.6° in the navigation group and 10.8° ± 6.5° in the control group (*p* < 0.001). The percentage of cups inside the Lewinnek safe zone was 93% in the navigation group and 44% in the control group (*p* < 0.001). The mean absolute values of navigation error were 3.3° ± 2.8° in inclination and 5.8° ± 4.9° in anteversion. Among the cases of osteoarthritis, the inclination error was significantly higher in Crowe group 2 to 4 than in Crowe group 1 (5.1° ± 3.5° and 3.0° ± 2.5°, respectively, *p* < 0.05). The percentage of hips with inclination error over 10° in Crowe group 2 to 4 was significantly higher than in Crowe group 1 (17 and 1%, respectively, *p* < 0.05).

**Conclusions:**

The image-free navigation system improved the accuracy of cup orientation. The accuracy of cup position was less in Crowe group 2 to 4 than in Crowe group 1.

## Background

In total hip arthroplasty (THA), correct acetabular component position is an important factor in preventing postoperative complications such as dislocation [1], impingement [2], accelerated polyethylene wear [3], and component loosening [4]. Lewinnek et al. [1] recommended an inclination angle of 40° ± 10° and an anteversion angle of 15° ± 10° as the safe zone for cup orientation in THA. The conventional technique using mechanical guides to determine acetabular component position intraoperatively has resulted in inaccurate cup orientation, even when performed by experienced surgeons [5, 6]. Intraoperative navigation systems, either computed tomography (CT)-based navigation systems or image-free navigation systems, have been shown to improve the accuracy of acetabular component orientation in THA [7].

In image-free navigation systems, the three-dimensional geometry of the pelvis (anterior pelvic plane; APP) is determined by anatomical landmarks acquired intra-operatively using a reference pointer. For cup implantation, alignment of the implant is calculated relative to the APP. Additional intra-operative image acquisition is not required.

In patients with developmental dysplasia of the hip (DDH), not every patient has the same acetabular morphology. It is not clear whether the severity of the hip deformity affects the accuracy of cup orientation in THA using image-free navigation systems.

The purpose of this study was to investigate the accuracy of acetabular cup placement in primary THA using an image-free navigation system and compare it to that of THA with conventional technique. Furthermore, we investigate the relationship of the accuracy of cup placement using image-free navigation system and the severity of the hip deformity.

## Materials and methods

From February 2017 to March 2020, 107 consecutive hips in 97 patients underwent primary cementless THA using an image-free navigation system (Brainlab Hip 6.0, Brainlab, Feldkirchen, Germany). The surgical approach was the posterior approach with the patient in the lateral position (85 hips) or the anterolateral supine approach in the supine position (22 hips). All patients in navigation group had a SQRUM TT SHELL (Kyocera, Osaka, Japan). As a control group, 77 retrospective consecutive hips in 64 patients who underwent THAs with manual implant-techniques between February 2012 and April 2017 were included. The surgical approach was the posterior approach with the patient in the lateral position (68 hips) or the anterolateral supine approach in the supine position (9 hips). A Regenerex Ringloc Acetabular Component was used in 39 hips, a Continuum Acetabular Shell was used in 28 hips, a G7 PPS Finned BoneMaster Acetabular Shell was used in 8 hip, Trilogy Acetabular Shell was used in 1 hip, and Trabecular Metal Acetabular Shell in 1 hip (all components were from Zimmer Inc., Warsaw, IN). The patients’ demographic characteristics are shown in Table 1.

**Table 1:** Demographic characteristics

	Navigation group	Control group	*P*
Gender (women/men)	87/20	70/7	ns
Age (years)	73.1 (45 ~ 89)	61.0 (34 ~ 86)	< 0.001
Body mass index (kg/m2)	23.9 (15 ~ 50)	23.5 (15 ~ 37)	ns
Diagnosis			
Osteoarthritis	92	70	ns
Crowe group 1	74	58	
Crowe group 2	13	8	
Crowe group 3	4	3	
Crowe group 4	1	1	
Osteonecrosis of the femoral head	7	7	
Rapidly destructive coxarthrosis	4	0	
Rheumatoid arthritis	2	0	
Trauma	2	0	
Surgical approach			
Posterior	85	68	ns
Anterolateral supine	22	9	

**Fig. 1:**
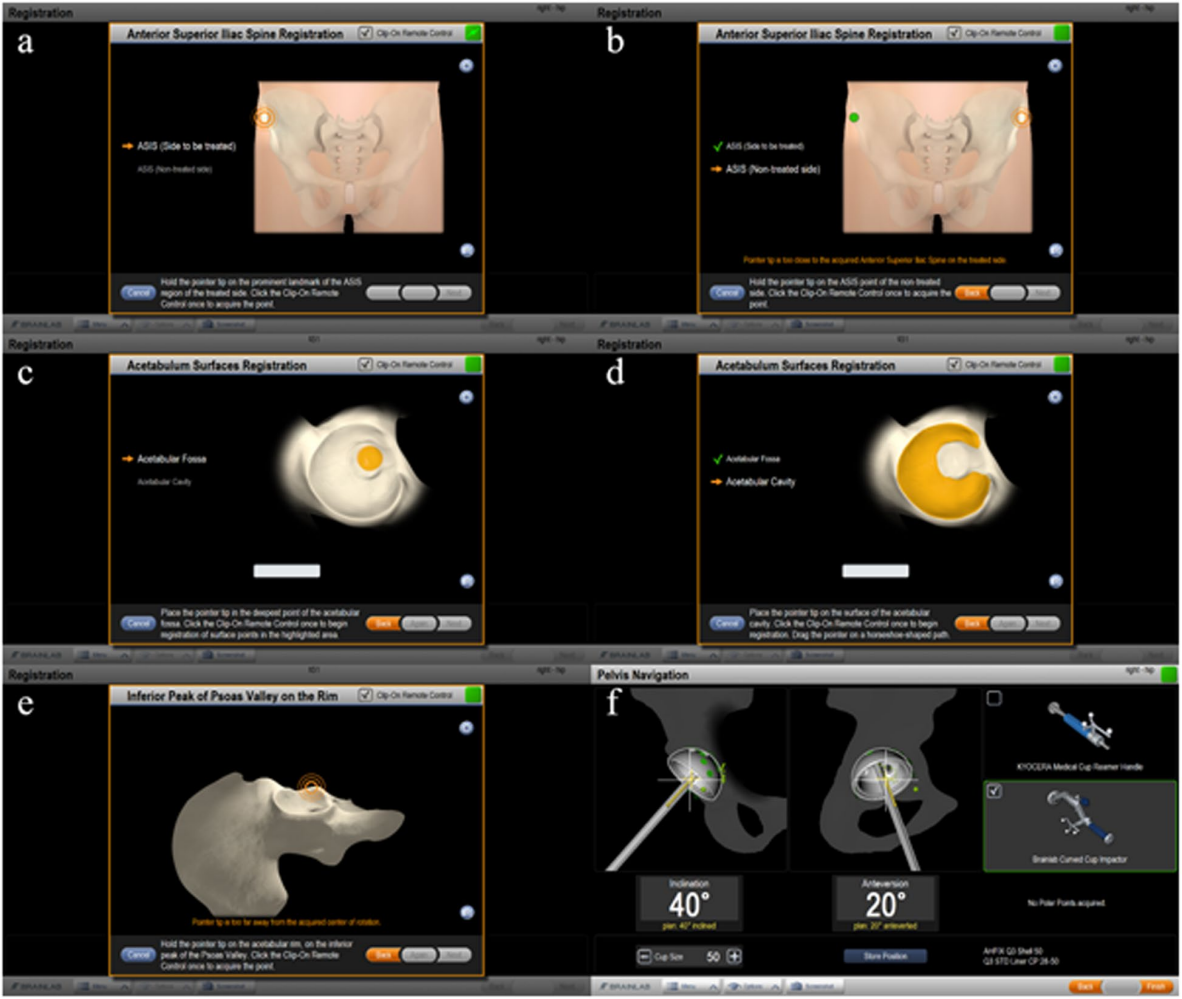
Image-free navigation system in this study. Registration of the bilateral anterior superior iliac spines (**a**, **b**). Registration of the acetabular fossa, the acetabular cavity, and the anterior rim point (**c**-**e**). Intraoperative measurment of the cup insertion angle (**e**)

Image-free navigation relies on an APP defined by the three bony landmarks (bilateral anterior superior iliac spines (ASISs) and the pubic tubercle). At the start of the surgery, a reference array was fixated into the iliac crest on the treated side approximately 3 cm proximal to the ASIS using two, 4-mm Schanz screws. The treated ASIS and non-treated ASIS were registered using a reference pointer (Fig. 1a, b). In the lateral position, this procedure was performed in a semi-sterile environment (the area around the pelvic reference array was draped, whereas the other areas remained non-sterile). In the supine position, this procedure was performed in a sterile environment. The acetabular fossa and the acetabular cavity were digitized (Fig. 1c, d). Additionally, a point located directly at the anterior side of the acetabular rim (anterior rim point) was acquired instead of registration of the pubic tubercle (Fig. 1e). During the procedure, cup operative anteversion and inclination angles were calculated relative to the APP. Cup orientation was planned to be operative inclination of 40° and operative anteversion of 20° (radiographic inclination of 42° and radiographic anteversion of 15°) relative to the functional pelvic plane (FPP) taking preoperative pelvic tilt into account (Fig. 1d). In the control group, because cup placement was performed using mechanical guide, target angle of cup operative inclination was 40° and anteversion was 20° (radiographic inclination of 42° and radiographic anteversion of 15°).

Postoperative cup position was assessed using a 3D-template system (ZedHip, Lexi, Tokyo, Japan) after CT examination. In this measurement, cup radiographic inclination and radiographic anteversion were evaluated relative to the FPP based on the definitions of Murray [8]. The absolute values of errors of radiographic inclination and radiographic anteversion were calculated by subtracting postoperative angles from the target angles (postoperative CT measurement–preoperative target angle) with respect to the FPP. The proportions of patients within the Lewinnek safe zone (40° ± 10° inclination; 15° ± 10° anteversion) were also assessed. To analyze the accuracy of the navigation system, the absolute differences between the intraoperative values measured by the image-free navigation system and the postoperative values measured by postoperative CT were calculated.

All patients were followed after THA, and complications were examined.

On the basis of previous data, the difference (mean ± standard deviation) between the image-free navigation and conventional groups of cup anteversion was 3.2 ± 3.0° [9]. The power calculation indicated that 14 cases would be necessary for the study relative to historical controls (α = 0.05, power = 0.8). The intraclass correlation coefficient (ICC) was used to analyze intra-observer and inter-observer reliabilities. All statistical analyses were performed using SPSS version 27 software (SPSS Inc., Chicago, IL). Patients’ demographic characteristics including age and body mass index (BMI) were compared between the two groups using the Mann–Whitney U test. The Chi-squared test and Fisher’s exact test were used to compare sex, diagnosis. The Mann–Whitney U test was used to compare the absolute values of errors. The Chi-squared test and Fisher’s exact test were used to compare the percentage of the hips within safe zone and the incidence of complication. Spearman’s rank correlation coefficients were used for correlation analysis between absolute values of navigation error (postoperative CT–navigation record) and BMI, the Mann-Whitney U test was used to compare the navigation error between women and men, between Crowe group 1 and Crowe group 2 to 4, and between the posterior approach and the anterolateral approach [10]. The Chi-squared test and Fisher’s exact test were used to compare the percentage of the hips over 10° of navigation error.

This study was approved by the institutional review board of our hospital, and informed consent was obtained from each patient.

## Results

**Fig. 2:**
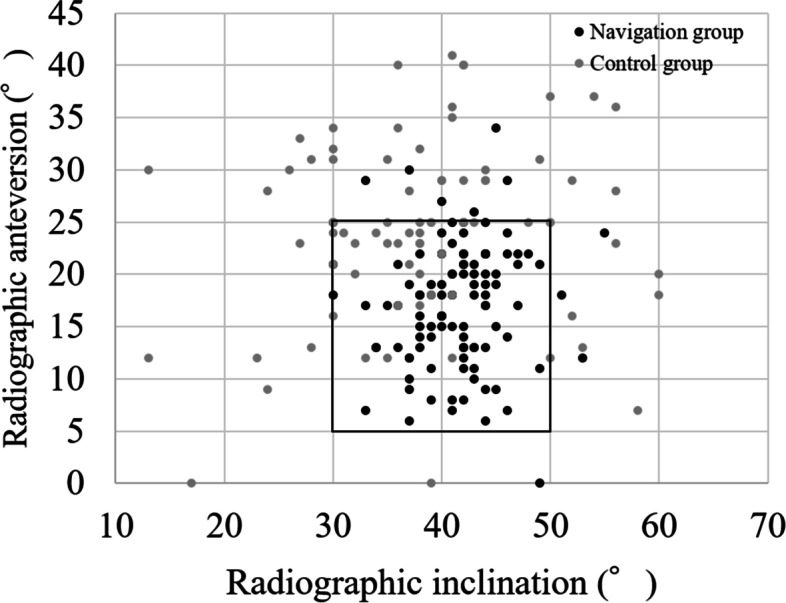
Positions of the acetabular component relative to Lewinnek safe zone. The percentage of cups inside the safe zone in the navigation group was significantly higher than in the control group (93 and 44%, respectively, *p* < 0.001)

The intra-observer reliabilities were 0.984 and 0.961 for inclination and anteversion, respectively. The inter-observer reliabilities were 0.962 and 0.947 for inclination and anteversion, respectively. The mean postoperative radiographic inclination relative to the FPP was 41.2° ±4.8° (range 26°–55°), and radiographic anteversion was 16.6° ±5.6° (range 0°-34°) in the navigation group. The mean postoperative radiographic inclination was 38.5° ±10.1° (range 13°–60°), and radiographic anteversion was 24.3° ±8.8° (range 0°-41°) in the control group. The mean absolute errors of the postoperative measured angles from the target angles in navigation group were 3.4° ± 3.0° (range 0°–13°) in inclination and 5.1° ± 3.6° (range 0°–19°) in anteversion. The mean absolute errors in control group were 8.4° ± 6.6° (range 0°–29°) in inclination and 10.8° ± 6.5° (range 1°–26°) in anteversion. There were significant differences in both the inclination error and the anteversion error between the two groups (*p* < 0.001 and *p* < 0.001, respectively) (Table 2). The percentage of cups inside the Lewinnek safe zone was 93% in the navigation group and 44% in the control group (*p* < 0.001) (Fig. 2). The mean absolute values of navigation error were 3.3° ± 2.8° (range 0°–12°) in inclination and 5.8° ± 4.9° (range 0°–26°) in anteversion (Table 3). The percentages of hips with error over 5° were 20% in inclination and 45% in anteversion. The percentage of hips with error over 10° was 4% in inclination and 11% in anteversion.

**Table 2:** Results of absolute values of errors of the measured postoperative angles from the target angles

	Navigation group	Control group	*P*
Inclination	3.4° ± 3.0°	8.4° ± 6.6°	< 0.001
Anteversion	5.1° ± 3.6°	10.8° ± 6.5°	< 0.001

**Table 3:** Results of absolute values of navigation error

Inclination	3.3° ± 2.8°
Anteversion	5.8° ± 4.9°

**Fig. 3:**
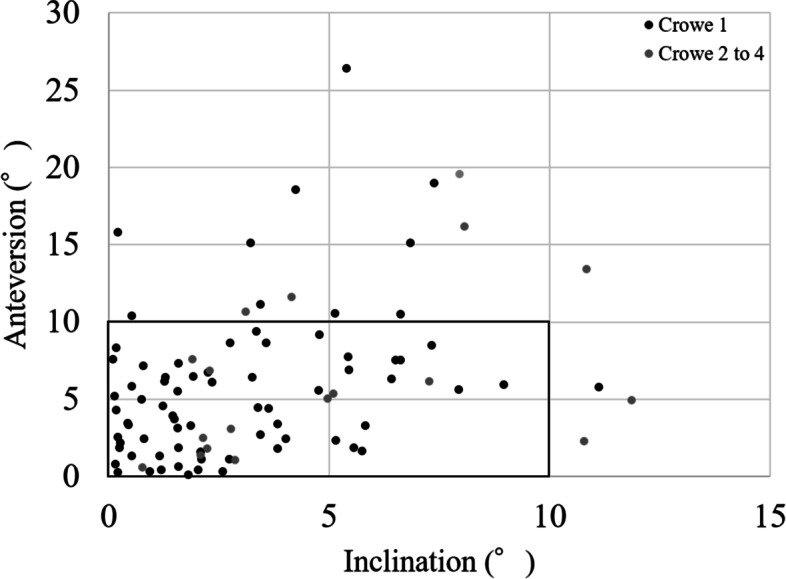
Scatterplot of navigation error of Crowe group 1 and Crowe group 2 to 4. The percentage of hips with inclination error over 10° was significantly higher in Crowe group 2 to 4 than in Crowe group 1

No significant correlations were observed between the mean absolute values of navigation error and BMI (inclination, R = 0.079, *p* = 0.421; anteversion, R = − 0.068, *p* = 0.485). There was no significant difference between the mean absolute values of women and men (*p* = 0.629 in inclination and *p* = 0.093 in anteversion). The mean absolute values of navigation error of Crowe group 1 were 3.0° ± 2.5° (range 0°–11°) in inclination and 5.8° ± 4.9° (range 0°–26°) in anteversion. The mean absolute values of navigation error of Crowe group 2 to 4 were 5.1° ± 3.5° (range 1°–12°) in inclination and 6.7° ± 5.5° (range 1°–20°) in anteversion. The inclination error was significantly higher in Crowe group 2 to 4 than in Crowe group 1 (*p* < 0.05), but there was no significant difference between the two groups in anteversion error (*p* = 0.653) (Table 4). The percentages of hips with error over 10° were 1% in inclination and 12% in anteversion in Crowe group 1, and 17% in inclination and 28% in anteversion in Crowe group 2 to 4. The percentage was significantly higher in Crowe group 2 to 4 than in Crowe group 1 in inclination (*p* < 0.05) (Fig. 3). The mean absolute values of navigation error of the posterior approach were 3.4° ± 2.8° (range 0°–12°) in inclination and 5.8° ± 5.1° (range 0°–26°) in anteversion. The mean absolute values of navigation error of the anterolateral supine approach were 2.7° ± 2.7° (range 0°–9°) in inclination and 5.7° ± 4.2° (range 0°–16°) in anteversion. There were no significant differences between the two groups (inclination, *p* = 0.279; anteversion, *p* = 0.739).

**Table 4:** Comparison of navigation error in Crowe group 1 and Crowe group 2 to 4

	Crowe group 1	Crowe group 2 to 4	*P*
Inclination	3.0° ± 2.5°	5.1° ± 3.5°	< 0.05
Anteversion	5.8° ± 4.9°	6.7° ± 5.5°	ns

As for complications, postoperative dislocation occurred in 1 case (0.9%) in the navigation group. In this case, the cup alignment was accurate (40° radiographic inclination and 19° radiographic anteversion), and revision THA was not performed. In the control group, postoperative recurrent dislocation occurred in 1 case (1.3%). In this case, the cup position was outside the safe zone, and the revision THA was performed. There were no significant differences in the incidence of dislocation between the two groups (*p* = 1.000). There were no complications related to the navigation procedures, such as pin site infection or nerve injury.

## Discussion

In the present study, the percentage of cups inside the Lewinnek safe zone was significantly higher in THA using an image-free navigation system than using conventional technique. Previous studies reported that the percentages of cups inside the Lewinnek safe zone ranged from 43 to 73% with conventional freehand arthroplasty [9, 11–13], 80 to 93% with an image-free navigation system [9, 12, 13], and 83 to 100% with a CT-based navigation system [11, 12]. These computer-assisted systems were shown to improve the accuracy of cup orientation, and the present results were comparable to those of previous studies. The mean absolute values of navigation error in the present study were 3.3° ± 2.8° in inclination and 5.8° ± 4.9° in anteversion. With image-free navigation systems, the inclination errors were reported to range from 2.9° to 3.7°, and the anteversion errors ranged from 4.2° to 6.8° [9, 12, 14]. With CT-based navigation systems, the absolute values of inclination errors were reported to range from 1.2° to 3.2°, and the anteversion errors ranged from 1.0° to 3.3° [12, 15–19]. The results for navigation errors in the present study were comparable to those of previous studies of image-free navigation systems (Table 5).

**Table 5:** Summary of computer-assisted navigation systems in total hip arthroplasty

		Lewinnek safe zone	Navigation error		Dislocation rate
			Inclination	Anteversion	
Image-free navigation	Kalteis et al. 2006 [12]	93%	2.9° ± 2.2°	4.2° ± 3.3°	0.0%
	Parratte et al. 2007 [13]	80%			0.0%
	Lass et al. 2014 [9]	90%	3.2° ± 2.4°	6.5° ± 3.7°	
	Takeda et al. 2017 [14]		3.7° ± 2.7°	6.8° ± 3.6°	0.0%
	Present study	93%	3.3° ± 2.8°	5.8° ± 4.9°	0.9%
CT-based navigation	Kalteis et al. 2006 [12]	83%	3.0° ± 2.6°	3.3° ± 2.3°	0.0%
	Murphy et al. 2006 [20]				0.5%
	Sugano et al. 2012 [11]	100%			0.0%
	Iwana et al. 2013 [16]		1.8° ± 1.6°	1.2° ± 1.1°	
	Tsutsui et al. 2017 [17]		1.5° ± 1.3°	2.1° ± 1.8°	
	Nakahara et al. 2018 [18]		1.2° ± 3.3°	1.0° ± 2.4°	
	Tetsunaga et al. 2020 [15]		2.7° ± 2.0°	2.8° ± 2.6°	0.0%
	Tetsunaga et al. 2020 [19]		3.2° ± 2.4°	3.0° ± 2.5°	0.0%

Ueoka et al. reported that the alignment of the acetabular component even for Crowe group 4 was as good as that for Crowe group 1 using a CT-based navigation system. The absolute mean deviations between the intraoperative and postoperative records were 1.2° ± 0.8° (inclination) and 1.4° ± 1.0° (anteversion) in Crowe group 4 and 1.3° ± 0.9° (inclination) and 1.4° ± 1.0° (anteversion) in Crowe group 1, with no significant differences between the 2 groups. In addition, no cup angle deviations, in either group, were greater than 5° [21]. In the present study, the inclination error was significantly higher in Crowe group 2 to 4 than in Crowe group 1, and the percentage of hips with inclination error over 10° were significantly higher in Crowe group 2 to 4 than in Crowe group 1. The present study showed that the accuracy of cup orientation was less in inclination for Crowe group 2 to 4 than for Crowe group 1 with an image-free navigation system. The reconstruction of the three-dimensional APP based on a point acquired on the ASISs and the acetabulum might be less accurate in Crowe group 2 to 4. To the best of our knowledge, this is the first report investigating the relationship between the accuracy of an image-free navigation system and the Crowe classification.

Lass et al. reported that no significant difference was found in implantation accuracy of an image-free navigation system between patients with BMI < 27 kg/m^2^ and ≥ 27 kg/m^2^. The mean postoperative inclination was 38.3° ± 4.0° in patients with a BMI < 27 kg/m^2^ and 38.1° ± 4.8° in the other group. The mean postoperative anteversion was 19.7° ± 8.2° in patients with a BMI < 27 kg/m^2^ and 17.2° ± 7.9° in the other group [9]. Takeda et al. reported that there were no significant differences between the obese (BMI ≥ 25 kg/m^2^) and non-obese (BMI < 25 kg/m^2^) groups for the absolute discrepancy between the intraoperative values and the postoperative values of cup inclination (3.8° ± 2.7° in the obese group and 3.7 ± 2.7° in the non-obese group) and anteversion (6.8° ± 3.4° in the obese group and 6.8° ± 3.6° in the non-obese group) with the image-free navigation system [14]. Similarly, there were no correlations between BMI and the absolute navigation errors either in inclination or anteversion in the present study.

Takeda et al. reported that there were no significant differences between the lateral position and supine position groups in the absolute discrepancy between the intraoperative values and the postoperative values for cup inclination (3.6° ± 2.6° in the lateral group and 3.8° ± 2.7° in the supine group) and anteversion (6.7° ± 3.6° in the lateral group and 7.1° ± 3.5° in the supine group) with an image-free navigation system [14]. Similarly, there were no significant differences between the lateral position and the supine position in the present study. One of the possible reasons is that the same landmarks were used for registration, and the APP was reconstructed in either the lateral position or the supine position.

Previous studies reported that the dislocation rate of THA ranged from 0 to 8% with the conventional freehand technique [11–13], 0% with image-free navigation systems [12–14], and 0 to 0.5% with CT-based navigation systems [11, 12, 15, 19, 20]. Intraoperative navigation systems were shown to be useful for reducing the incidence of postoperative dislocation. In this study, although postoperative dislocation was occurred in one case (0.9%), the cup position was inside the safe zone (Table 5). Although the accuracy of cup placement was improved significantly, there was no significant difference in dislocation rate between navigation group and control group. From a cost-to-benefit perspective, it is not clear whether image-free navigation system is necessary in every standard hip replacement patient, or should be limited to more technically demanding cases to reduce dislocation rate. Long-term follow-up is necessary to analyze the cost utility of using image-free navigation system to reduce dislocation rate.

The present study has some limitations. First, clinical results were not investigated except for complications such as dislocation. Further studies are needed to show the clinical advantages of image-free navigation. Second, the mean BMI of 23.8 kg/m^2^ in this study was relatively lower than that in American or European patients. However, in previous report of European patients, the mean BMI was 27.6 kg/m^2^, and the BMI was not related to the accuracy of an image-free navigation system [9].

## Conclusions

The image-free navigation system improved the accuracy of cup orientation in THA. The accuracy of cup insertion was less in Crowe group 2 to 4 than in Crowe group 1.

## Data Availability

The datasets during and/or analyzed during the current study are available from the corresponding author on reasonable request.

## Abbreviations

THA: Total hip arthroplasty
CT: Computed tomography
APP: Anterior pelvic plane
DDH: Developmental dysplasia of the hip
ASISs: Anterior superior iliac spines
FPP: Functional pelvic plane
ICC: Intraclass correlation coefficient
BMI: Body mass index
